# Revisiting Non-Conventional Crystallinity-Induced Effects on Molecular Mobility in Sustainable Diblock Copolymers of Poly(propylene adipate) and Polylactide

**DOI:** 10.3390/molecules27217449

**Published:** 2022-11-02

**Authors:** Panagiotis A. Klonos, Alexandra Evangelopoulou, Zoi Terzopoulou, Alexandra Zamboulis, Miguel Ángel Valera, Ana Mangas, Apostolos Kyritsis, Dimitrios N. Bikiaris

**Affiliations:** 1 Department of Physics, National Technical University of Athens (NTUA), Zografou Campus, 15780 Athens, Greece; 2 Laboratory of Polymer Chemistry and Technology, Department of Chemistry, Aristotle University of Thessaloniki, 54124 Thessaloniki, Greece; 3 AIMPLAS, Asociación de Investigación de Materiales Plásticos Y Conexas, Carrer de Gustave Eiffel, 4, 46980 Valencia, Spain

**Keywords:** diblock copolymers, polylactide, poly(propylene adipate), crystallization, molecular mobility

## Abstract

This work deals with molecular mobility in renewable block copolymers based on polylactide (PLA) and poly(propylene adipate) (PPAd). In particular, we assess non-trivial effects on the mobility arising from the implementation of crystallization. Differential scanning calorimetry, polarized light microscopy and broadband dielectric spectroscopy were employed in combination for this study. The materials were subjected to various thermal treatments aiming at the manipulation of crystallization, namely, fast and slow cooling, isothermal melt- and cold-crystallization. Subsequently, we evaluated the changes recorded in the overall thermal behavior, semicrystalline morphology and molecular mobility (segmental and local). The molecular dynamics map for neat PPAd is presented here for the first time. Unexpectedly, the glass transition temperature, *T*_g_, in the amorphous state drops upon crystallization by 8–50 K. The drop becomes stronger with the increase in the PPAd fraction. Compared to the amorphous state, crystallization leads to significantly faster segmental dynamics with severely suppressed cooperativity. For the PLA/PPAd copolymers, the effects are systematically stronger in the cold- as compared to the melt-crystallization, whereas the opposite happens for neat PLA. The local *β*_PLA_ relaxation of PLA was, interestingly, recorded to almost vanish upon crystallization. This suggests that the corresponding molecular groups (carbonyl) are strongly involved and immobilized within the semicrystalline regions. The overall results suggest the involvement of either spatial nanoconfinement imposed on the mobile chains within the inter-crystal amorphous areas and/or a crystallization-driven effect of nanophase separation. The latter phase separation seems to be at the origins of the significant discrepancy recorded between the calorimetric and dielectric recordings on *T*_g_ in the copolymers. Once again, compared to more conventional techniques such as calorimetry, dielectric spectroscopy was proved a powerful and quite sensitive tool in recording such effects as well as in providing indirect indications for the polymer chains’ topology.

## 1. Introduction

Polymers are all over the world, from everyday life uses to industrial and space applications [1,2]. This is mainly due to the combination of unique properties of polymers (high performance), the relatively easy processing and low economic cost of production. During the last decades, there has been a significant growth in environmental concerns, which, in the case of polymers, is reflected in the oil-based molecular origin of the most traditional polymers. Additionally, the polymers’ accumulation in nature is of great concern nowadays, since they exhibit extremely low/slow degradability [3,4,5]. Thus, the scientific community has dedicated much attention to more eco-friendly materials, including polymers, which are mainly bio-based and can be synthesized from renewable resources. The family of polylactides (PLA) has, during the last decade, become the most commercially viable polymer [6,7,8,9,10,11]. PLAs are aliphatic polyesters synthesized from a renewable product of the fermentation of agricultural compounds, i.e., the lactic acid [12], mainly via the ring opening polymerization, ROP [13,14]. PLA exhibits a glass transition temperature, *T*_g_, closely above the room temperature, whereas it can be both amorphous and semicrystalline [10,15,16]. These characteristics are strongly connected within both processing and performance (mechanical strength, thermal conductivity, permeation of small molecules) [10,15,17,18,19,20], with the potential to manipulate the latter. Crystallinity is the number one factor involved in the performance and macroscopic properties. To be more precise, the crystalline fraction, CF, as well as the semicrystalline morphology, the crystals’ dense or sparce distribution and the number/size/density of the crystallites, are the key parameters [21,22,23,24]. Strongly connected to the crystallinity, nucleation and crystal growth is the mobility of PLA chains, more precisely, the diffusion capability of the chains (cooperativity, chain–chain entanglements) and folding (chain rigidity) [25,26].

Apart from the good properties of PLA, this polyester is lacking in the rate of physical biodegradation, as the latter demands very long time periods for physical biodegradation via enzymatic hydrolysis [25,27,28]. This is due to the existence of strong ester bonds that make the mechanism of hydrolytic scission quite ineffective [25,29]. As expected, this problem can be bypassed via chemistry. An efficient route to improving the biodegradability of PLA-based systems and, at the same time, preserving the desired performance and even creating new properties, is the combination of PLA with other sustainable polymers. In this frame, numerous PLA-based copolymers, polymer blends and interpenetrating polymer networks have been synthesized [30,31,32,33,34,35].

Following this approach, we have employed a relatively new class of polyesters, poly(*n*-alkylene adipates), PnAAd, and prepared a series of copolymers and blends with PLA [30,31,32,33]. PnAAd belong to the family of non-toxic aliphatic polyesters that can also be semicrystalline [36,37]. The synthesis of PnAAd is based on adipic acid, a substance that can be prepared from renewable resources [38], while it is listed among the most important dicarboxylic acids in the International Energy Agency. PnAAd serve well as ‘improving’ means for other polyesters, owing to three characteristics, i.e., the low *T*_g_ of PnAAd [33,37,39,40,41], combined with high rates of biodegradation along with good thermal stability [40]. In addition, the polar terminal groups of PnAAd (mainly −OH) favor the use of PnAAd as initiators for the ROP of PLA [30], which is quite convenient from the chemistry point of view, both from the laboratory as well as the upper-scale production. In particular, the low *T*_g_ and the relatively flexible polymer chain make these polyesters good candidates to serve as ‘plasticizers’ for PLA [26,30,31,32,33], resulting in, among other outcomes, the improvement of the biodegradation and compostability of the PLA/PnAAd systems (copolymers and blends).

The present article reports a more ‘basic research’-targeted investigation. In our recent work on diblock copolymers with poly(propylene adipate) (PPAd) and PLA forming the two blocks [26], we recorded interesting and non-trivial findings with respect to molecular mobility. We recorded that in the amorphous state, the increase in the PPAd fraction led to an expected lowering of *T*_g_, whereas, strikingly, the involvement of non-isothermal crystallization resulted in a further suppression of *T*_g_ (even below 0 °C). The crystallinity-indued suppression of *T*_g_ was systematically facilitated with the PPAd fraction. The phenomenon was surprising as the existence of crystals is expected, in general, to hinder the polymer chains mobility and, consequently, to elevate *T*_g_. Herein, we attempt to check this behavior, and to further follow the effect by more carefully selected crystallization protocols. We employ the combination of differential scanning calorimetry (DSC), polarized light microscopy (PLM) and broadband dielectric spectroscopy (BDS) to study the effects on segmental mobility in PLA/PPAd blends as compared to neat PLA and initial PPAd. The investigation mainly involves the implementation of three states regarding crystallinity, namely, amorphous, isothermally melt-crystallized and isothermally cold-crystallized. Finally, we construct the corresponding molecular mobility (dynamics) map for these cases. This is the first recording of the molecular dynamics for initial PPAd, to the best of our knowledge.

## 2. Experimental

### 2.1. Materials

**Figure 1:**
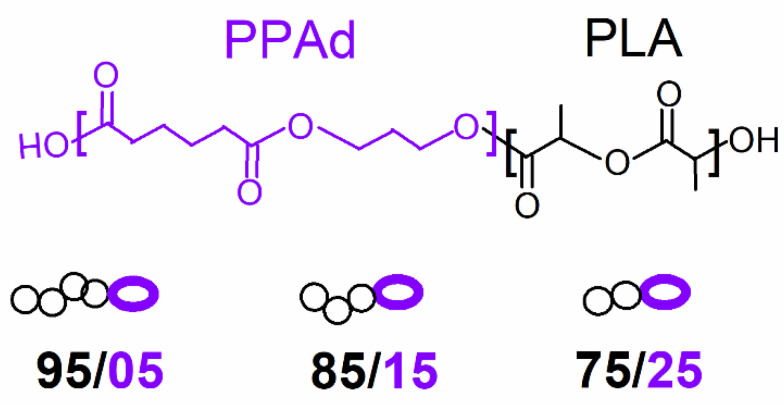
Chemical structure of PPAd and PLA diblock copolymers under investigation.

The materials investigated here are diblock copolymers based on PLA and PPAd, synthesized in a previous work by Terzopoulou et al. [30]. Briefly, the copolymers were prepared by using an initial PPAd polymer of low *M*_n_ ~ 6 kg/mol, forming the first block, onto which the second block of PLA was build, in situ, via ring opening polymerization of L-lactide at 180 °C (Figure 1). The samples differ in the mass ratio PLA (%)/PPAd (%) as 95/5, 85/15 and 75/25 and are listed in Table 1 along with values on the estimated average molar masses (*M*_n_). As reference samples, we comparatively study the initial PPAd (the same as that used in the copolymer preparation) and a neat PLA prepared by a similar ROP route (Table 1). Regarding the initial scope of these copolymers, this was a success as the enzymatic degradation of PLA is significantly accelerated in the copolymers as compared to PLA in bulk.

### 2.2. Experimental Methods

#### 2.2.1. Differential Scanning Calorimetry

**Figure 2:**
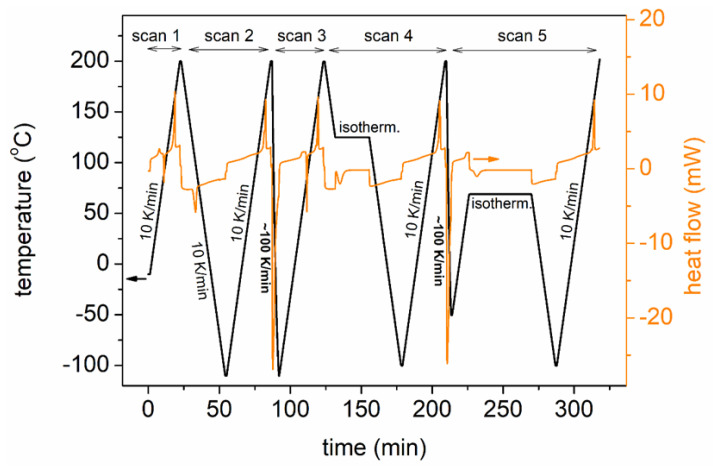
(Left axis, black line) The time–temperature profiles for Scans 1–5 of the DSC measurements. (Right axis, orange line) A representative example of the recorded heat flow against time is shown.

The glass transition and crystallization of the block copolymers as well as initial PLA and PPAd were assessed by DSC. To that aim, we employed a TA Q200 calorimeter (TA, New Castle, DE, USA), combined with a liquid nitrogen control system. The instrument had been calibrated with indium for temperature and enthalpy and sapphires for heat capacity. The thermograms were recorded in N_2_ atmosphere of high purity (99.995%) and within the range from −110 to 200 °C. In total, five (5) scans were performed, schematically described in Figure 2, first involving a heating scan for erasing thermal history (Scan 1), and four main scans aiming at manipulating crystallization. In particular, in Scan 2, the melted samples were cooled at 10 K/min, in Scan 3, the melted samples were cooled at the highest achievable rate in order to eliminate crystallization, while in Scans 4 and 5, the samples were subjected to isothermal melt- and cold-crystallization annealings at selected temperatures (*T*_anneal,mc_ and *T*_anneal,cc_, respectively), different for each sample and chosen based on the results of the previous Scans 2 and 3. Details on the selection of *T*_anneal_ are given along with the experimental results. The values for *T*_anneal_ are listed in Table 1. Upon each crystallization treatment, the sample was cooled to −110 °C and subsequently a final heating scan was recorded. The heating rate was fixed for all scans at 10 K/min.

The characteristic temperature of the glass transition step, *T*_g_, was estimated from the heating curve as the point of half increase in the heat capacity. Crystallization and melting events were evaluated in terms of peak temperature maxima, onsets and enthalpy changes (Δ*H* in J/g). The crystalline fraction, CF, was estimated by both the melt- and cold-crystallization peaks, both isothermals and non-isothermals, and by comparing the crystallization enthalpy (Δ*H*, respectively) with the theoretical heat of fusion for a 100% crystalline PLA, Δ*H*_100%,PLA_, usually taken as 93 J/g [42] according to Equation (1).

(1) $$\frac{\Delta H}{\Delta H_{100\%,\mathrm{PLA}}}$$

Please note that, more recently, compared to the work by Fischer et al. [42], there more alternative values for Δ*H*_100%,PLA_ have been reported, considering the individual crystal polymorphs of PLA (*α* and *α′*) [43,44].

#### 2.2.2. Polarized Light Microscopy

The PLM technique was employed to follow the effect of the copolymer composition on the semicrystalline morphology. PLM micrographs were recorded isothermally during melt- and cold-crystallization at the same *T*_anneal,mc_ and *T*_anneal,cc_ as those employed in DSC (Table 1). The micrographs were recorded by means of a Nikon Optiphot-1 polarizing microscope equipped with a Linkam THMS 600 heated stage, a Linkam TP91 control unit and a Jenoptik Gryphax Arktur camera.

From the data of PLM, the spherulitic growth rate was followed during isothermal melt-crystallization, at the same *T*_anneal,mc_ as those in DSC. In the isothermal crystallization step, a minimum of three spherulites were followed during their free growth before they impinged on one another. Then, the radius of each spherulite was measured and plotted as a function of time. From this plot, the slope represents the spherulitic growth rate (*G*) at the selected *T*_anneal,mc_. The latter estimation was technically impossible for the case of cold-crystallization.

#### 2.2.3. Broadband Dielectric Spectroscopy

The molecular mobility, with emphasis on segmental mobility, was investigated by BDS [45], employing a Novocontrol BDS setup (Novocontrol GmbH, Montabaur, Germany). Pieces of the produced sample were initially placed and melted between finely polished brash electrodes of 14 mm in diameter. Silica spacers of ~100 μm in thickness were used in order to prevent the electrical contact of the electrodes and keep them parallel with each other. Consistent with the above techniques, three thermal protocols were adopted for BDS, namely, preparing (1) amorphous samples by melting and fast cooling, (2) isothermally melt-crystallized samples at *T*_anneal,mc_ and (3) isothermally cold-crystallized samples at *T*_anneal,cc_. For each sample and protocol, the complex dielectric permittivity, *ε″* (Equation (2)), was recorded in N_2_(g) nitrogen flow, isothermally as a function of frequency, *f*, in the range from 10^–1^ to 10^6^ Hz and in the temperature range between −150 and 120 °C, upon heating in steps of 5 and 10 K.

(2) $$\varepsilon^{*}\left(f,T\right)=\varepsilon^{\prime }\left(f,T\right)-i\cdot \varepsilon \prime \prime \left(f,T\right)$$

The permittivity spectra are mainly complex as they consist of multiple contributions (relaxation mechanisms). Therefore, the spectra were analysed by the fitting of special models, mainly, the Havriliak–Negami, HN [45,46], function (Equation (3)).

(3) $$\varepsilon^{*}\left(f\right)=\varepsilon_{\infty }+\frac{\Delta \varepsilon }{{\left[1+{\left(if/f_{0}\right)}^{\alpha_{\mathrm{HN}}}\right]}^{\beta_{\mathrm{HN}}}}\;$$

Therein, ε_∞_ describes the value of the real part of dielectric permittivity, *ε′*, for *f* >> *f*_0_, Δ*ε* is the dielectric strength, *f*_0_ is a characteristic frequency related to the frequency of maximum dielectric loss and *α*_HN_ and *β*_HN_ are shape parameters, for width and symmetry, respectively. Upon this analysis, we constructed the timescale map of local and segmental relaxations. The local processes usually obey the Arrhenius equation [45,47] (Equation (4)),

(4) $$f\left(T\right)=f_{0,\mathrm{Arrh}}\cdot e^{-\frac{E_{\mathrm{act}}}{kT}}\;$$

as they exhibit a temperature-independent activation energy, *E*_act_. On the other hand, the segmental relaxations, related to the glass transition, demonstrate a different timescale due to their cooperative character, usually described by the Vogel–Fulcher–Tammann–Hesse (VFTH) expression [45,48] (Equation (5)),

(5) $$f\left(T\right)=f_{0,\mathrm{VFTH}}\cdot e^{-\;\frac{DT_{0}}{T-T_{0}}}$$

within which, *D* is the so-called fragility strength parameter [48] and is related to the measure of cooperativity, namely, the fragility index, *m* (Equation (6)).

(6) m=16+590/D

## 3. Results and Discussion

### 3.1. Crystallization and Glass Transition

**Figure 3:**
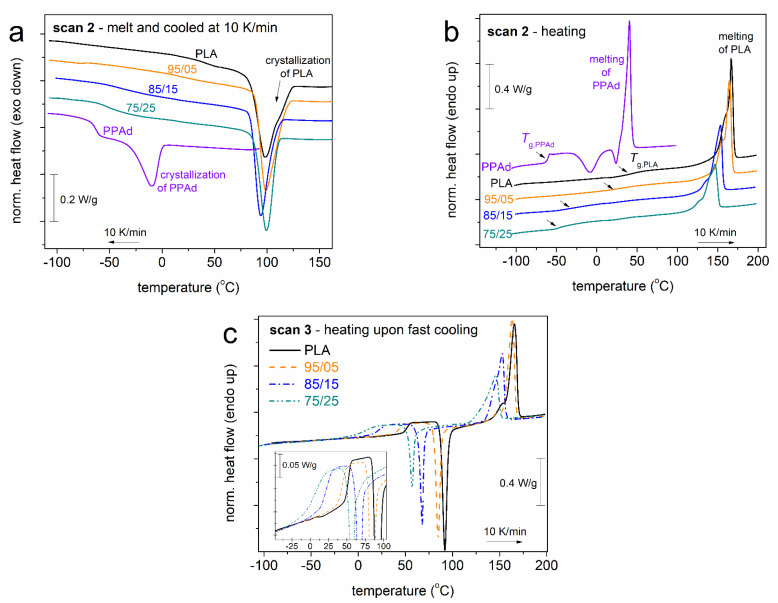
Comparative DSC traces during (**a**) cooling and (**b**) heating at 10 K/min of Scan 2 and (**c**) heating at 10 K/min for all initially amorphous samples previously cooled at a high rate of Scan 3. The recorded heat flow (in mW) is shown upon normalization to the sample mass (W/g). The inset to (**c**) shows the glass transition region in more detail.

In Figure 3, we present the calorimetric results for Scans 2 and 3, upon erasing any thermal history. During cooling at 10 K/min (Figure 3a), all copolymers crystallize at ~100 °C, similarly to neat PLA. Initial PPAd crystallizes at −10 °C, exhibiting an enthalpy change of ~21 J/g. During the subsequent heating at 10 K/min, PPAd exhibits a *T*_g_ of −61 °C. The corresponding heat capacity change Δ*c*_p_ = 0.42 J/g∙K. On the other hand, the *T*_g_ of neat PLA equals 43 °C. The addition of PPAd in the copolymers results in a decrease in the *T*_g_ from 39 °C down to the quite low value of −42 °C. This suppression, along with the absence of a second glass transition step in the copolymers, suggests the homogeneity (no significant micro-phase separation) of the copolymers, which was actually the goal of said synthesis.

The results shown in Figure 3b involve the effects of the copolymer composition as well as the effects of crystallinity on *T*_g_. Therefore, to assess the direct effects of composition on *T*_g_, we performed measurements on amorphous samples. These are shown in Figure 3c, i.e., via the heating scan recorded upon a prior fast cooling. With the increase in PPAd content from 0 to 25%, the glass transition temperature decreases from 51 down to 11 °C. The drop in *T*_g_ of PLA by the addition of PPAd on the same polymer chain was rationalized in terms of the plasticization effect of the small PPAd blocks. The effect is also facilitated by the overall shortening of the copolymer chains [26]. The surprising effect is the further lowering of *T*_g_ with the implementation of crystallinity, as, conversely, the presence of crystals would be expected to hinder the polymer chains diffusion and, subsequently, to elevate the *T*_g_ [16,49,50,51] (and references therein). So far, these results confirm previous recordings on the same systems [26], which actually generated the interest for the present ‘follow-up’ study.

**Figure 4:**
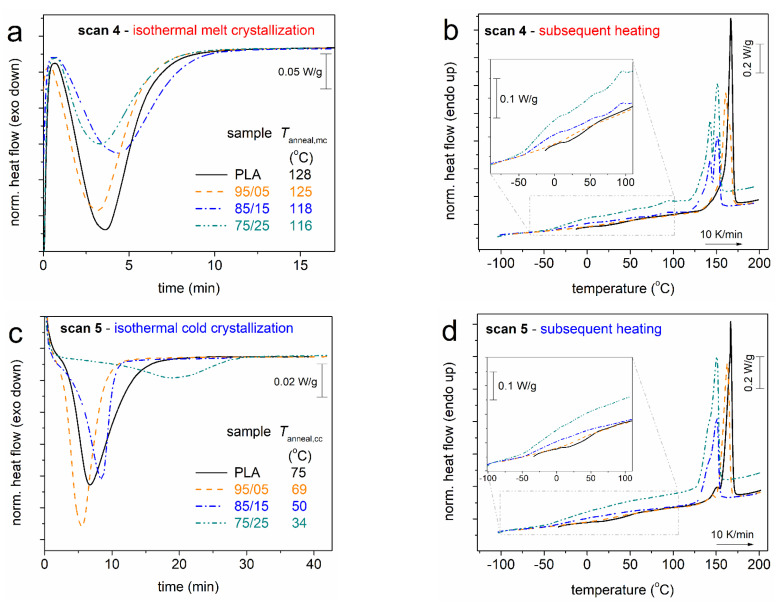
Comparative DSC traces of (**a**,**b**) Scan 4 and (**c**,**d**) Scan 5, for PLA and PLA/PPAd copolymers in terms of (**a**,**c**) isothermal crystallization and (**b**,**d**) the subsequent heating. The heat flow curves have been normalized to each sample mass. The insets to (**b**,**d**) show details in the temperature range of glass transition.

Based on the results of Scan 2 (Figure 3a), in particular, from the temperature range wherein the sample is neither melted nor has crystallization begun, we have selected suitable temperatures (Table 1) to perform the isothermal melt-crystallization annealing, *T*_anneal,mc_. Then, based on the results of Scan 3 (Figure 3c), we have chosen suitable *T* to perform the cold-isothermal crystallization annealings, *T*_anneal,cc_ (Table 1), namely, to be above *T*_g_ and below the event of cold-crystallization. In particular, the *T*_anneal_ values were chosen as the *T* just before (3–5 K) the initiation of each non-isothermal crystallization event. The corresponding DSC results are shown in Figure 4.

In Figure 4a,c, we present the time evolutions of crystallization, while in Figure 4b,d, we show the subsequent heating scans. Almost all systems, including neat PLA, exhibit similar crystallization rates. The exception to this behaviour is the cold-crystallization of 75/25, which is somewhat retarded. The effects are actually due to the different *T*_anneal_ selected for the different samples, thus, the result on the crystallization rate should not be compared between the different samples for drawing conclusions on the direct effect of the copolymeric structure on the nucleation and crystal growth (e.g., the performance of Avrami analysis [52]). For the most clear conclusions, the results of Figure 4 (Scans 4 and 5) have been evaluated and are discussed in terms of crystalline fraction (CF, Equation (1)) and the overall results (Scans 2–5) in terms of characteristic temperatures, namely, crystallization, *T*_c_, onset of crystallization, *T*_c,onset_, cold-crystallization, *T*_cc_, onset of cold-crystallization, *T*_c,onset_, and glass transition temperature. Please note that the *T*_g_ is always estimated from the corresponding heating scan.

It is important to note that, based on the results of Figure 3a, the temperature range of the crystallization of the copolymers coincides to that of neat PLA, not PPAd. Therefore, we have concluded that the recorded crystallization should involve the PLA-rich phases. Thus, in Equation (1), the crystallization enthalpy (Δ*H*) used for calculations has been normalized to the PLA mass content, *w*_PLA_ (Δ*H*/*w*_PLA_). Otherwise, it would not be correct to compare the recorded Δ*H* to the heat of fusion of PLA.

**Figure 5:**
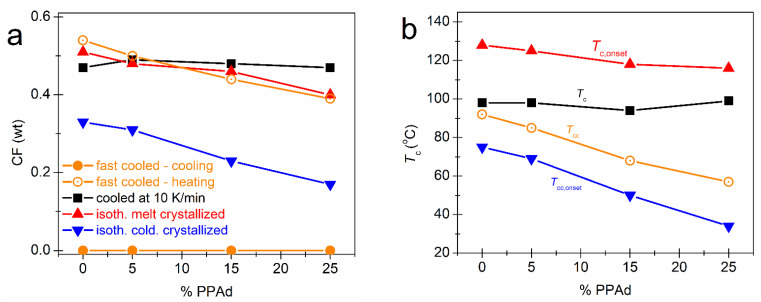
The PPAd content dependence of (**a**) the crystalline fraction and (**b**) crystallization temperatures (peaks or onsets), as evaluated from the various scans (thermal treatments) employed.

In Figure 5, we follow the effects of the PPAd loading and of each thermal treatment on CF. CF = 0 upon the fast cooling, which denotes that the cooling rate of ~100 K/min (in the temperature range of the expected crystallization) is sufficient to eliminate crystallization [21,23]. However, this rate is not sufficient to prevent nucleation, which is also expected for conventional cooling in PLA [23]. For Scans 3–5, in Figure 5a, CF drops monotonically by ~10% with the addition of PPAd. The same happens with the characteristic *T*s of crystallization (melt and cold) in Figure 5b, which drops by about 10–40 K (depending on the thermal treatment). The results suggest that both the amounts as well as the rates of crystallization (nucleation/lamellae packings) are slower/hindered in the copolymers. The retarded lamellae packing should have an impact on the quality (density and/or size) of the spherulites. This is partly confirmed by the lowering of the melting temperature, *T*_m_, with PPAd loading (see Figure 3b,c and Figure 4b,d). The situation seems to be more complex for the case of Scan 2 (non-isothermal crystallization), within which both the CF and *T*_c_ do not seem to vary significantly. In our previous work [26] on the same systems, we presented PLM results for the crystallization during the cooling of Scan 2 which showed non-systematic effects on the rate of crystallization (faster for PLA and 85/15 and slower for 95/05 and 75/25) while the final size of the spherulites was found to increase in the copolymers. Additionally, compared to most scans, the isothermal cold-crystallization has resulted in both lower CF and *T*_c_. This is most probably due to poor mobility of the chains during crystallization, which is reflected in the doubled (on average) crystallization times needed (Figure 4c), compared to the case of melt-crystallization.

**Figure 6:**
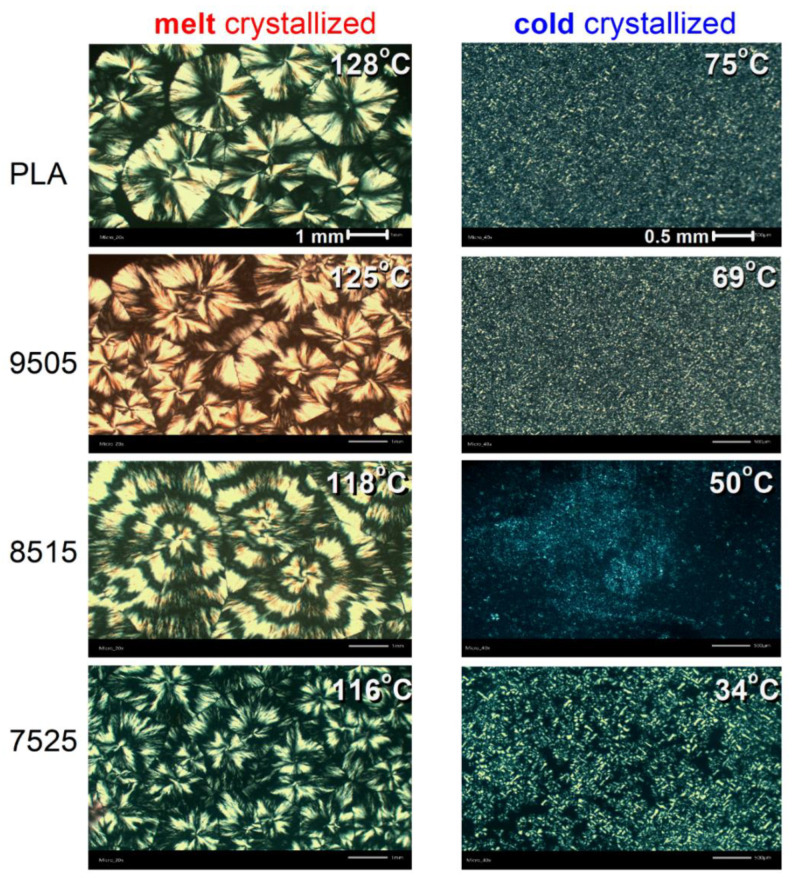
PLM micrographs for all the PLA-based samples suffered (**left**) melt- and (**right**) cold-isothermal crystallization, at the marked temperatures. The shown images correspond to the final states of crystallization. The added scale bars at the top -left and -right images correspond to all PLM images, i.e., 1 mm for the left-side and 0.5 mm for the right side.

**Figure 7:**
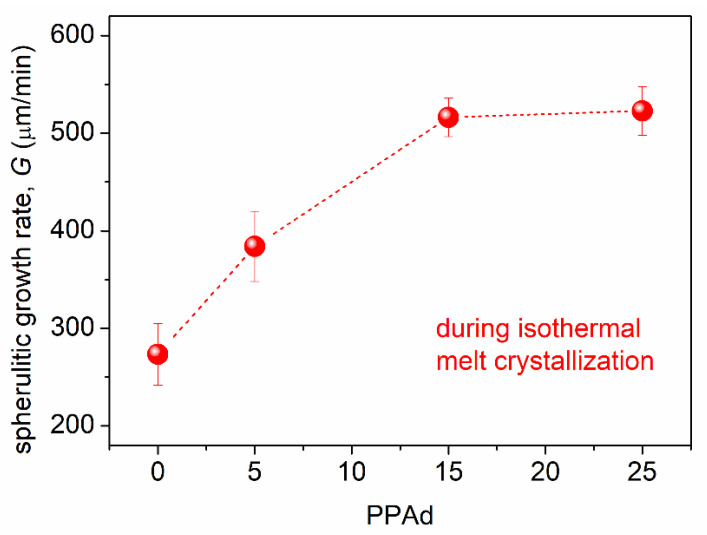
The PPAd dependence of the spherulitic growth rate evaluated during melt-crystallization of the PLA-based systems.

From the results of PLM (Figure 6) during melt-crystallization, we were able to estimate the spherulitic growth rate, *G*, which is presented in Figure 7. *G* increases in the copolymers from ~270 (neat PLA) up to ~520 μm/min (75/25) with the increase in PPAd. The effect could be correlated with the easier diffusion of the chains as manifested by the lowering of *T*_g_.

Regarding the semicrystalline morphology upon the isothermal cold-crystallization (right side of Figure 6), we could not conclude its to effects on the size of the formed spherulites. However, from a more careful look at the data and upon repeating the measurements on various spots and different samples, we observed that, contrary to PLA, 95/05 and 85/15, in the case of 75/25 the formed spherulites are smaller, and the sample volume is not completely filled with crystals. The latter also seems true in the case of melt-crystallization. We will come back at this point later. Finally, we observe for the copolymers that the spherulites exhibit the so called ‘ring-banded’ structure [53]. This is quite clear in 85/15 and 75/25. The phenomenon is expected when the polymer chains consist of both crystallizable and non-crystallizable segments and low *M*_n_, in general, such as in our case. Ring-banded spherulites have been observed for both PLA [54] and poly(butylene adipate) [55].

**Figure 8:**
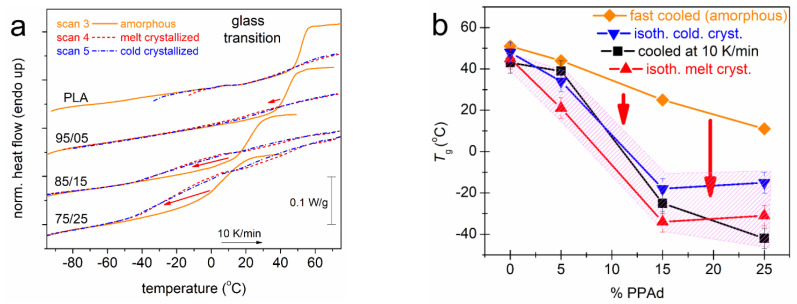
(**a**) Comparative DSC traces during heating showing the glass transition for all samples along with the changes imposed from the amorphous to the semicrystalline state (marked by the added arrows). (**b**) The PPAd fraction dependence of the glass transition temperatures, as recorded during heating by the various thermal treatments. The shaded area includes all the cases of semicrystalline polymers. The added vertical arrows mark the effects imposed on ‘amorphous state’ *T*_g_ by the implementation of crystallization.

We may come now to the most interesting effect recorded herein. The basis for the discussion of this is Figure 8. Therein, we have plotted together the heating curves of Scans 3, 4 and 5 (Figure 8a). The results clearly show that the glass transition step is sharp and strong (high Δ*c*_p_) in the case of the amorphous samples. Upon crystallization (both types), the glass transition becomes more broad and weaker, which is expected, nevertheless, it migrates toward lower temperatures. In terms of *T*_g_, in Figure 8b, it is shown that whereas in the amorphous state the addition of PPAd leads to a maximum drop of *T*_g_ by ~40 K (75/25), **the implementation of crystallinity additionally lowers the *T*_g_ by 8 K in PLA, ~15 K in 95/05 and ~50 K in 85/15 and 75/25**. The effect seems controversial considering the expected effects of crystallinity, usually imposed on conventional polymers, hindering the mobility of the chains and elevating the *T*_g_ [16,49,50,51].

Before attempting to provide physically rational explanations for this, we will discuss the results in terms of molecular dynamics obtained via BDS and the corresponding critical analysis.

### 3.2. Molecular Mobility (BDS)

**Figure 9:**
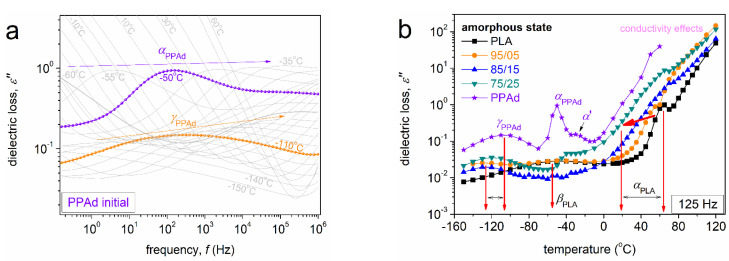
(**a**) Raw BDS spectra in terms of isothermal *ε″*(*f*) curves shown at the example of initial PPAd. (**b**) Comparative isochronal curves of *ε″* against temperature for all samples, upon replotting from the raw isothermal curves, for the initial amorphous state. The results in (**b**) correspond to the representative frequency of 125 Hz. The main recorded relaxation mechanisms, recorded as peaks of *ε″*, are indicated on the plots. The added red arrow in (**b**) marks the effect on the main relaxation imposed by the increase of the PPAd fraction.

**Figure 10:**
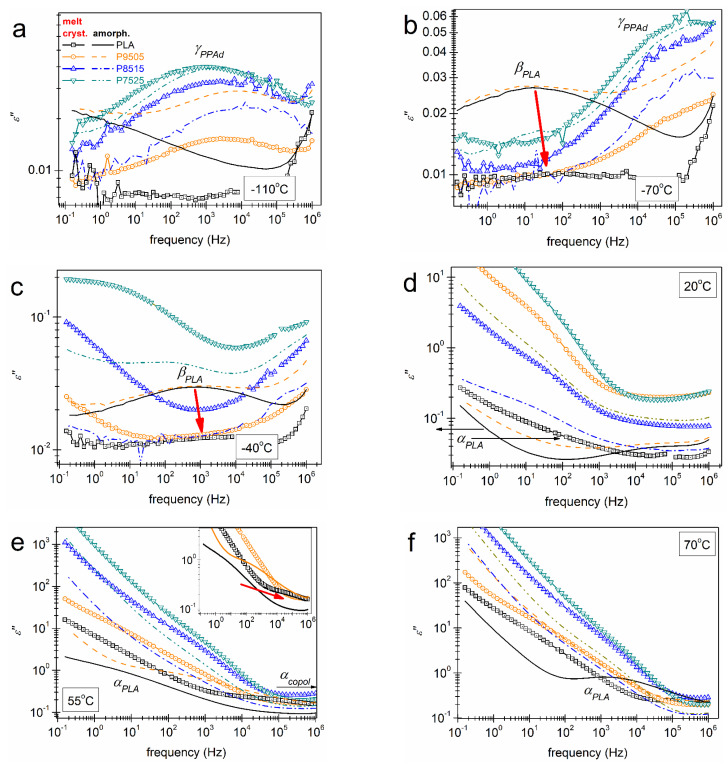
Isothermal spectra of *ε″*(*f*) plots for all copolymers and neat PLA shown comparatively at various temperatures (indicated on the plots). The lines correspond to samples in the amorphous state, namely, melted and fast-cooled prior to the measurement, while the symbols connected with lines correspond to samples that had isothermally melt-crystallized at *T*_anneal,mc_ prior to the BDS measurement. The added red arrows mark the effects imposed by the implementation of crystallinity.

**Figure 11:**
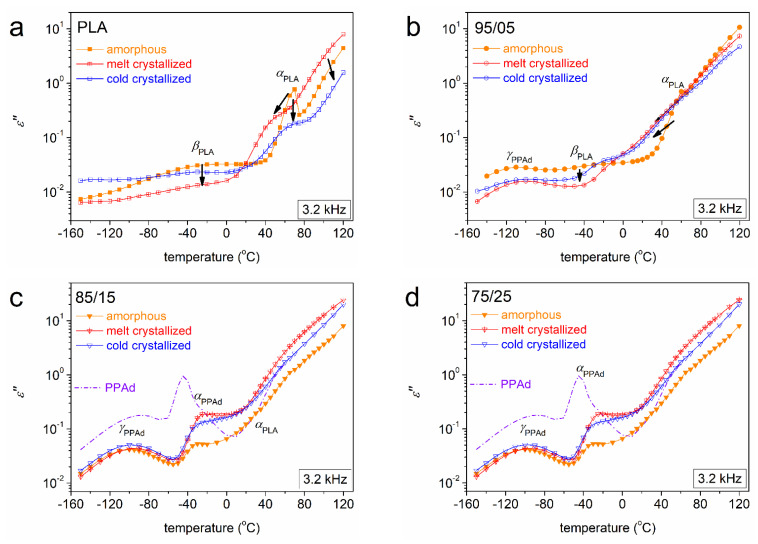
Comparative isochronal *ε″*(*T*) plots at *f* ~ 3.2 kHz for (**a**) PLA, (**b**) 95/05, (**c**) 85/15 and (**d**) 75/25, showing the effects on relaxation processes from the amorphous state (solid symbols) to the semicrystalline state (crossed and open symbols). For comparison, we added in the *ε″*(*T*) plot at *f* ~ 3.2 kHz for initial PPAd in (**c**,**d**). The added arrows mark the crystallinity-imposed effects.

In Figure 9, Figure 10 and Figure 11, we present raw BDS data in various forms. The molecular mobility is usually assessed in BDS by following the imaginary part of the dielectric permittivity, *ε″*, which is considered to be related with the dielectric loss [45,56]. An example of raw *ε″*(*f*) is shown in Figure 9a for initial PPAd. At *T* < *T*_g_ the dipolar relaxation mechanisms recorded as peaks in *ε″*(*f*) are considered to arise from local molecular motions of corresponding polar groups. These secondary relaxation mechanisms are generally named as *β*, *γ*, *δ*, etc. [45]. Then, as *T* increases and approaches *T*_g_, the dielectric signal increases by one or more orders of magnitude and the strong main relaxation enters the frequency window. This is the case of the dielectric analogue of glass transition usually called ‘*α* relaxation’, as it monitors the segmental mobility of the polymer chains via the relaxation of the dipoles perpendicularly distributed on the main polymer chain [45].

In Figure 9a, we can follow the local *γ*_PPAd_ located between 10^2^ and 10^3^ Hz at −110 °C and the segmental *α*_PPAd_ located between ~10^2^ and 10^3^ Hz at −50 °C by the naked eye for PPAd. The relaxation peaks migrate toward higher frequencies upon increasing temperature (increasing the provided thermal energy) due to acceleration of the corresponding molecular groups. To facilitate a more direct comparison with calorimetry, the raw BDS data (isothermal curves, Figure 9a and Figure 10) can be presented upon replotting to the form of ‘isochronal’ *ε″*(*T*) curves. These are shown in Figure 9b for all samples in the initially amorphous state and at *f* ~ 125 Hz. Therein, PLA exhibits a local and a segmental relaxation, *β*_PLA_ and *α*_PLA_, respectively.

Regarding the situation is the copolymers, the recorded relaxations can be followed in the isothermal (Figure 10) and isochronal (Figure 11) plots, that are used for comparisons both between the different copolymer compositions and amorphous-semicrystalline states.

Τhe copolymers’ spectra are more complex than those of the individual homopolymers, we have performed critical analysis of the spectra and constructed the molecular mobility maps. These maps show the timescale for all relaxations (i.e., dynamics), in terms of the peak frequency maxima, log*f*_max_, against the inverse temperature, 1000/*T* (otherwise called Arrhenius plots).

**Figure 12:**
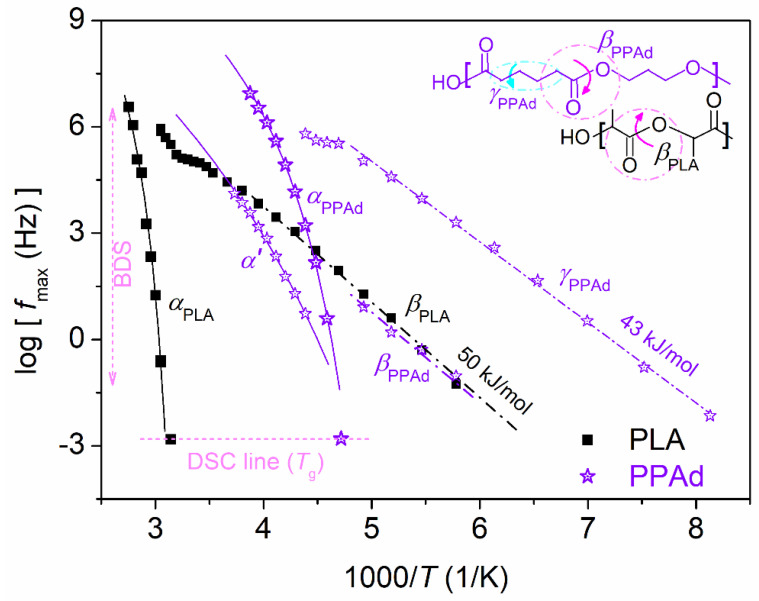
Arrhenius plots (dielectric relaxation map) for neat amorphous PLA and initial amorphous PPAd. The line connecting the experimental points are fittings of the VFTH (curved solid lines) and the Arrhenius (straight dash-dotted lines) equations. The calorimetric glass transition points have been added for comparison at the equivalent frequency of DSC.

In Figure 12, we present the overall maps for the initial PPAd and neat amorphous PLA. Therein, for PLA, *β*_PLA_ is shown, a local process originating from fluctuations or twisting motions of the –C=O group at the backbone of PLA (inset to Figure 12) [57,58,59]. *β*_PLA_ exhibits linear behavior (obeying the Arrhenius law) and an activation energy of ~50 kJ/mol. At higher temperature, the *α*_PLA_ is recorded, with its timescale points well-fitted with the VFTH equation (curved line in Figure 12), denoting the cooperative character of the main relaxation. The fragility index for *α*_PLA_ was estimated *m*_α_ = 178. Please also note that the good agreement between the BDS points on *α*_PLA_ relaxation and the calorimetric *T*_g_ (=51 °C, DSC line in Figure 12). From the BDS points, we may estimate the ‘dielectric glass transition temperature’ as the point where the extrapolation of the VFTH fitting meets the equivalent frequency of DSC, *f*_eq_, with log*f*_eq_ ~ −2.8, as the relaxation time in DSC is ~100 s. This way, *T*_g,diel_ was estimated as ~50 °C for PLA.

To the best of our knowledge, the full range timescale map for PPAd is presented here for the first time. In Figure 12, the fastest relaxation is *γ*_PPAd_, which is expected to screen the most localized mobility of the polymer. Unfortunately, there are still limited data in the literature on the dynamics of these relatively new class of polymers. By simply comparing the structure-dynamics of PPAd with other polymers exhibiting partial similarities in their structure, such as poly(ethylene glycol)s, poly(*n*-alkylene acrylate)s [60,61,62,63], we have suggested [26] that *γ*_PPAd_ could arise from the crankshaft motions of methylene sequences at the chain backbone (inset scheme to Figure 12). *γ*_PPAd_ follows the Arrhenius trend and the corresponding *E*_act_ is ~43 kJ/mol. At higher temperatures, another local-like was revealed, only via the fitting, as the process is quite weak. Due to the latter, this relaxation could not be resolved (safely) within the copolymers [26]. This process is named *β*_PPAd_. The timescale points of *β*_PPAd_ are almost identical to those for *β*_PLA_. Therefore, we suspect that it has similar molecular origins, as PLA and PPAd both carry backbone –C=O groups (Figure 12, inset scheme). Results from previous works on the similar polymer, poly(butylene adipate) [32,39], provide support for the proposed origins for *β*_PPAd_. At *T* ≥ *T*_g_, the segmental *α*_PPAd_ is recorded. We should report, from the methodological point of view, that *α*_PPAd_ was fitted by an asymmetric HN term (Equation (3)) with *α*_HN_ ~ 0.6–0.7 and *β*_HN_ ~ 0.6. As in the case of neat PLA, the dielectric and calorimetric *Τ*_g_ values, respectively, −62 and −61 °C, are also quite alike in terms of initial PPAd. Finally, another more retarded and weaker relaxation located close to *α*_PPAd_ was resolved. The relaxation can also be identified by the naked eye, e.g., in Figure 9b and Figure 11c,d, as a shoulder of *α*_PPAd_. The process could be fitted by a symmetric HN term (*β*_HN_ = 1) with *α*_HN_ ~ 0.4–0.6. In Figure 12, its timescale denotes cooperative character, whereas its extrapolation to the *f*_eq_ of DSC meets the region of the calorimetry *T*_g_. These facts denote that this process depends on segmental mobility and is either coupled with *α*_PPAd_ or a modified version of *α*_PPAd_. Thus, the process is named here as *α′*. Recalling the low *M*_n_ ~ 6 kg/mol of neat PPAd, this ‘coupling’ of *α*_PPAd_ and *α′*, resembles the situation between the main relaxations and the so-called Normal Mode relaxation [64,65,66]. Normal Mode arises from the fluctuation of the polymer chain end-to-end vector [57]. The relaxation is mainly recordable for short polymer chains (low *M*_n_) such as those in our case. However, Normal Mode relaxations are generally stronger and narrower (higher *α*_HN_) than *α′*. More work is needed to shed light on the molecular origins of *α′*.

At this point, we turn the focus onto the copolymers. The changes imposed on local dynamics in the PLA/PPAd copolymers have been discussed in our previous work [26]. The main focus here is on the main dynamics. Before that, however, we should focus on *β*_PLA_. In Figure 10b,d and Figure 11a, *β*_PLA_ is quite strong in the amorphous state. Upon melt-crystallization, strikingly, the relaxation is almost eliminated. This suggests that the corresponding molecular group (backbone carbonyl) is strongly involved and immobilized within the semicrystalline regions. Interestingly, upon cold-crystallization, *β*_PLA_ is suppressed as compared to the amorphous state, however, to a lesser extent than the melt-crystallized state. This difference can be rationalized by the lower CF_cc_ as compared to CF_mc_ (Figure 5a) and/or by the expected looser lamellae packing in the case of cold-crystallization. In previous excellent works by Ezquerra and co-workers [39,67] who studied polyesters, including poly(butylene adipate), similar effects on the local relaxations were revealed, assigned to changes in the chain–chain associations also related to crystallinity.

**Figure 13:**
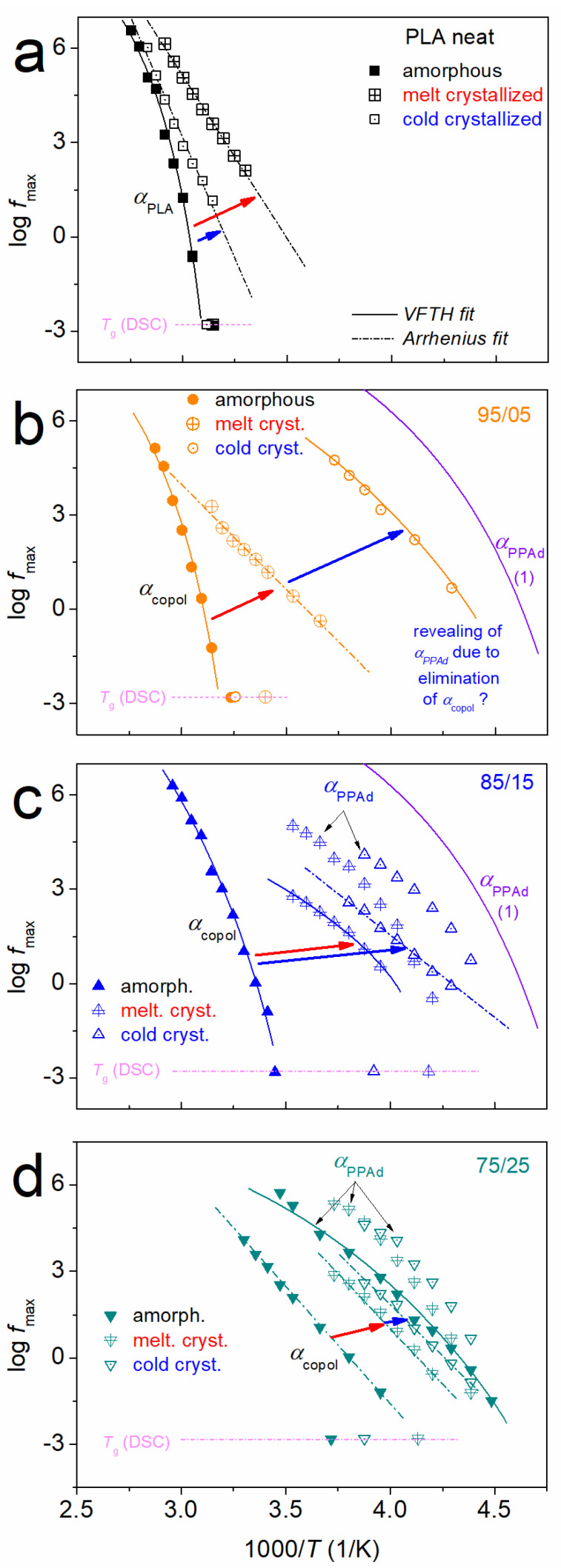
Arrhenius plots for (**a**) PLA, (**b**) 95/05, (**c**) 85/15 and (**d**) 75/25, showing the effects imposed on the segmental mobility (alpha relaxations) of initially amorphous samples by melt- and cold-crystallization (arrows). The added line (1) in (**b**,**c**) is the VFTH fitting result of *α*_PPAd_ of the initial PPAd.

Focusing on segmental mobility, we follow in the comparative dynamics maps of Figure 13, which at all cases shows that the involvement of crystallization leads to accelerated segmental mobility. In neat PLA (Figure 13a), cold-crystallization accelerates *α*_PLA_, while melt-crystallization accelerates it even more. Simultaneously, the cooperativity of *α*_PLA_ seems to vanish, as manifested by the transition from the VFTH behavior (amorphous) to linear Arrhenius-like behavior (semicrystalline). Qualitatively similar effects are recorded on the *α* relaxation in the copolymers, *α*_copol_, whereas an opposite impact as compared to neat PLA is recorded in the copolymers, within which, cold-crystallization leads to more extensive acceleration than the melt-crystallization. Obviously, the strength of the main relaxation is suppressed upon crystallization in all cases. Due to this strength suppression, we were able to distinguish double segmental dynamics in the semicrystalline copolymers 85/15 and 75/25. We recall that in the amorphous state, only 75/25 exhibited two relaxations, *α*_copol_ and *α*_PPAd_ [26]. This was evaluated as an indication of nanophase separation for 75/25 [26].

**Figure 14:**
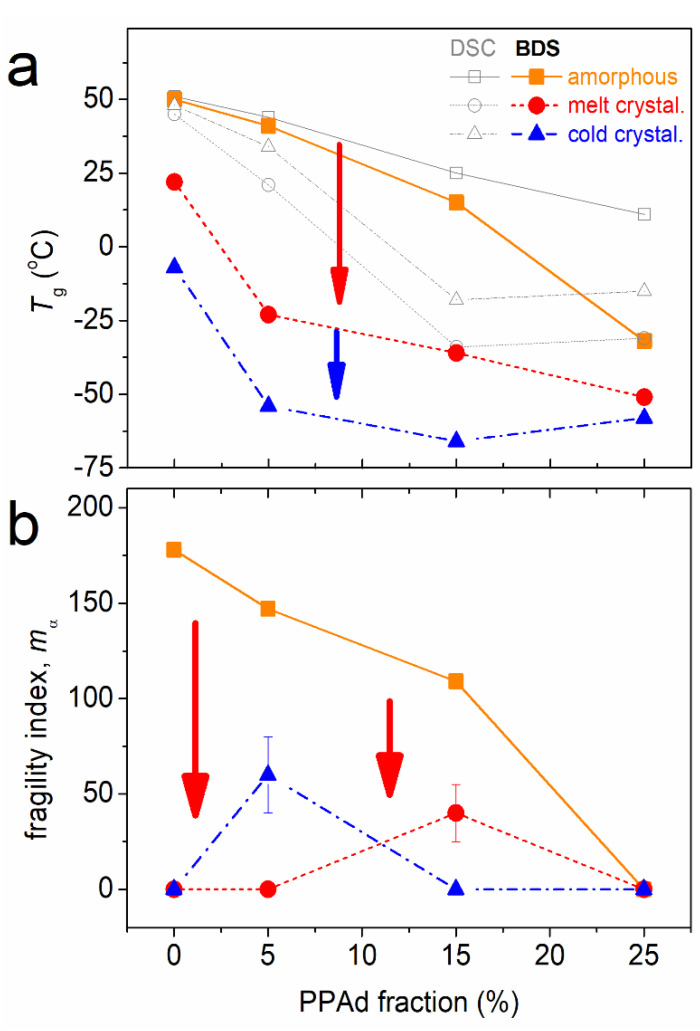
The PPAd fraction dependence of (**a**) the dielectric and calorimetric *T*_g_ and (**b**) fragility index of *α*_PLA_ relaxation, *m*_α_, for the different polymer states regarding crystallinity. The added arrows mark the crystallinity induced effects.

In Figure 14, we concentrate the overall dynamics data in terms of *T*_g,diel_ (Figure 14a) and fragility (cooperativity, Figure 14b). The impact of PPAd addition is systematically the acceleration in dynamics and suppression in fragility (mainly the vanishing of cooperativity). These effects are strongly enhanced, i.e., in the same direction, when crystallization is implemented.

These are non-trivial effects imposed by crystallization, at least for conventional polymers/homopolymers, as the presence of crystallites is considered a factor that tends to decelerate the segmental mobility [68].

We should keep in mind that, upon crystallization, the fraction of mobile amorphous PLA is decreasing. Thus, at first approximation, the plasticization effect of PPAd within the amorphous areas could be more pronounced, as compared to the fully amorphous state (Scan 3). One scenario that could further explain the overall data on mobility and crystallization is in terms of spatial confinements and constraints imposed by crystallization on the amorphous PLA and PLA–PPAd chains. The spatial confinement would have a strong impact when the amorphous zones formed between the crystallites are of dimensions comparable to the cooperativity length, *ξ*, [16,69] namely, some nanometers [70,71,72,73,74]. Values in such a situation cannot be easily checked, especially, the dimensions of the inter-chain distances in particular. The advanced structural characterization technique of small-angle X-ray scattering could provide further insight on this point [75,76].

The results shown in Figure 13 and Figure 14 can also be discussed from an alternative point of view for the copolymers. It is interesting that upon crystallization, the segmental dynamics tend to approach the fast dynamics of the initial PPAd. Considered together with the mobility maps, the shape parameters and the temperature dependences on *α*_copol_ (not shown), we suspect that upon crystallization, the mobility of PLA (i.e., the contribution of *α*_PLA_ to *α*_copol_) vanishes. The effect is stronger for cold-crystallization in the copolymers. Due to that, a weaker relaxation was revealed, the timescale of which resembles that of *α*_PPAd_. This could be the modified, decelerated, version of bulky *α*_PPAd_ in the copolymers [26,33]. According to this scenario, partly compatible with the previous one, the majority of PLA is ‘dielectrically immobilized’ within the formed crystals and both the local backbone groups and the overall chains are dielectrically inactive (vanishing of *β*_PLA_ and *α*_PLA_). In this context, the PLA-like segmental dynamics disappears and the ‘semicrystalline’ *α* relaxation is dominated by the PPAd-phases. This implies that crystallization of the PLA-rich phases leads to some kind of further phase separation of the PPAd segments, which are more active, at least dielectrically.

In this context, it is worthy to note that from the methodological point of view, the significant mismatch between the calorimetric and the dielectric *T*_g_ in the case of copolymers upon crystallization is shown in Figure 14a. Despite the general comment involving the ‘in principle’ different techniques that follow different modes (thermal events vs. dielectric relaxations) [56,59,77], we gain indications that, most probably, DSC is able to record the amorphous part of PLA (high calorimetric *T*_g_), whereas BDS is not. Thus, the *T*_g,diel_ in the semicrystalline state mainly follow the dynamics of PPAd (lower *T*_g_ in Figure 14a).

A final point worthy of discussion refers to the ionic conductivity effects in Figure 10 and Figure 11. Therein, it follows that a sharp increase in the dielectric signal is recorded at temperatures well above *T*_g_. As mentioned previously, this originates in the transportation of small charges (ions) throughout the sample. Obviously, the transportation of ions can take place only via the rubbery domains. In the copolymer, the ionic conductivity always dominates the signal at *T* > *T*_g_ of the copolymer. In none of the samples, amorphous or semicrystalline states, do we record a contribution of the ionic conductivity at lower temperatures, in particular at *T* > *T*_g_ of initial PPAd. This more macroscopic observation suggests, on the one hand, that the PLA–PPAd distribution is excellent, and, on the other hand, in any state, there is no continuity of the pure PPAd phase throughout the copolymer’s volume. A similar situation had been recorded in a previous work on PLA/poly(butylene adipate) diblock copolymers [32]. On the contrary, in polymeric blends of PLA and poly(ethylene adipate) (PEAd), exhibiting partial miscibility, we recorded significant continuous paths of PEAd throughout the copolymer volume, as manifested by strong ionic conductivity arising from PEAd [33].

## 4. Conclusions

Diblock copolymers of PPAd and PLA, prepared by ROP of lactic acid onto PPAd segments of low *M*_n_, are studied here regarding crystallization-induced effects on molecular mobility. In the amorphous state, the materials were found to be homogeneous with the PPAd playing a plasticization role on *T*_g_, systematically lowering with PPAd. Upon carefully chosen crystallization treatments, isothermal melt- and cold-crystallization, as well as non-isothermal crystallization, an interesting effect was revealed. *T*_g_ is significantly suppressed in the presence of crystals, by 8 to 50 K. This effect is, at first glance, controversial as the crystals usual hinder the chains’ mobility, leading to the elevation of the *T*_g_. The *T*_g_ drop was, interestingly, found to be facilitated by the increasing PPAd amount in the copolymers. For the highest amount of PPAd (25%), partial phase nano-separation was revealed, only by BDS [26], via the individual recording of the weak *α*_PPAd_ relaxation next to the bulk-like *α*_copol_. This is here proposed to be responsible for the formation of smaller crystals, not being able to completely fill the whole sample’s volume. The phase separation was also recorded upon crystallization, for 25% and 15% PPAd, as the PLA-originating dielectric response was suppressed overall, and that of PPAd rose ‘artificially’. Upon crystallization, the fragility index of segmental dynamics was found to severely decrease, and in some cases, to even vanish. The overall effects suggest the involvement of spatial nanoconfinement of the amorphous polymer between the spherulites or, in a more complex situation, involving additional PLA/PPAd separation driven by the crystal’s formation. Overall, crystallization seems to make PPAd the dominant polymer over PLA in terms of the mobility of the copolymers. This is found true for both the segmental and the local mobility, as, for example, the local *β*_PLA_ is quite strong in the amorphous state and almost vanishes upon crystallization. This scenario enabled the rationalizing of the significant discrepancy regarding *T*_g_ as recorded between DSC and BDS, with the dielectric *T*_g_ being lower in all copolymers than the calorimetric one.

## Figures and Tables

**Table 1:** The materials under investigation used the code names shown here, and the corresponding *M*_n_ values [30]. Included are the values for the selected temperatures of isothermal annealing of crystallization, *T*_anneal,mc_ and *T*_anneal,cc_, for melt- and cold-crystallization, respectively.

Sample	Code Name	*M*_n_ (g/mol)	*T*_anneal,mc_ (°C)	*T*_anneal,cc_ (°C)
PLA	PLA	76k	128	75
PLA (95%)_*b*_PPAd (5%)	95/05	63k	125	69
PLA (85%)_*b*_PPAd (15%)	85/15	41k	118	50
PLA (75%)_*b*_PPAd (25%)	75/25	29k	116	34
PPAd initial	PPAd	6k	-	-

## Data Availability

Not applicable.
